# Otoferlin Depletion Results in Abnormal Synaptic Ribbons and Altered Intracellular Calcium Levels in Zebrafish

**DOI:** 10.1038/s41598-019-50710-2

**Published:** 2019-10-03

**Authors:** Aayushi Manchanda, Paroma Chatterjee, Josephine A. Bonventre, Derik E. Haggard, Katie S. Kindt, Robert L. Tanguay, Colin P. Johnson

**Affiliations:** 1 Molecular and Cellular Biology Program, Oregon State University, Corvallis, Oregon USA; 2 Department of Biochemistry and Biophysics, Oregon State University, Corvallis, Oregon USA; 3 Department of Environmental and Molecular Toxicology, Oregon State University, Corvallis, Oregon USA; 4 National Institute of Deafness and Other Communication Disorders (NIDCD), NIH, Maryland, USA

**Keywords:** Hair cell, Molecular neuroscience

## Abstract

The protein otoferlin plays an essential role at the sensory hair cell synapse. Mutations in otoferlin result in deafness and depending on the species, mild to strong vestibular deficits. While studies in mouse models suggest a role for otoferlin in synaptic vesicle exocytosis and endocytosis, it is unclear whether these functions are conserved across species. To address this question, we characterized the impact of otoferlin depletion in zebrafish larvae and found defects in synaptic vesicle recycling, abnormal synaptic ribbons, and higher resting calcium concentrations in hair cells. We also observed abnormal expression of the calcium binding hair cell genes s100s and parvalbumin, as well as the nogo related proteins rtn4rl2a and rtn4rl2b. Exogenous otoferlin partially restored expression of genes affected by endogenous otoferlin depletion. Our results suggest that in addition to vesicle recycling, depletion of otoferlin disrupts resting calcium levels, alters synaptic ribbon architecture, and perturbs transcription of hair cells specific genes during zebrafish development.

## Introduction

Hair cells serve as the receptor cell for both the auditory and vestibular system of vertebrates^1,2^. In response to a pressure-induced deflection of stereocilia at the apical end of the cell, mechanosensitive ion channels open and alter the voltage of the cell membrane^3^. Depolarization of the cell membrane triggers opening of L-type voltage-gated calcium channels at the basolateral side of the hair cell which stimulates neurotransmitter release from presynaptic vesicles^4,5^. Stimulated vesicular release from hair cells in turn elicits action potentials in afferent neurons^6^. The graded potential generated by stereocilia deflection can induce exocytosis at rates that can reach several hundred vesicles per second and requires specialized molecular components^7^. Prominent among these specializations is the synaptic ribbon, a large electron dense organelle composed of several proteins including ribeye. The synaptic ribbon tethers vesicles proximal to the presynaptic membrane which is thought to facilitate high rates of sustained synaptic transmission^8–12^. Loss of ribeye results in depletion of synaptic ribbons and abnormal vesicle release, highlighting the importance of the ribbon in synaptic signaling^8,13,14^. Another defining component of the hair cell synapse is otoferlin. Otoferlin is a multi-C2 domain protein that functions in synaptic vesicle exocytosis and endocytosis^7^. A proposed role for otoferlin in conferring calcium sensitivity to exocytosis is based on several studies, including the finding that otoferlin knockout mice are profoundly deaf and do not release neurotransmitter from hair cells in response to calcium influx^15^. In addition, *in vitro* reconstitution studies indicate that otoferlin enhances the rate of liposome fusion in a calcium sensitive manner^16^. These *in vitro* studies demonstrated that mutation of the putative calcium binding residues in the C2C domain abrogates activity of the domain, and subsequent studies demonstrated a change in calcium sensitivity of exocytosis upon mutation of these residues *in vivo*^17^. Less well understood is whether the function of otoferlin is conserved across species. In addition, although the effects of otoferlin on mouse hair cell development have been reported, the generality of the findings and the developmental effects in other organisms have not been determined^15,18,19^.

We have previously validated zebrafish as a model to study otoferlin *in vivo*, and demonstrated that depletion of otoferlin results in reduced startle reflex and loss of balance, consistent with a role for otoferlin in neurotransmitter release^20^. In this study, we investigated the role of otoferlin in hair cell development by examining hair cells depleted of otoferlin at several developmental time points. We found that in addition to reduced synaptic vesicle recycling, loss of otoferlin also resulted in abnormal synaptic ribbons. Further, analysis of calcium imaging measurements indicated that resting calcium levels were elevated in otoferlin depleted neuromasts. We also examined the gene expression profile of zebrafish larvae lacking otoferlin and found altered expression of genes associated with calcium handling and neuronal growth. Our results indicate that loss of otoferlin disrupts intracellular calcium and neurotransmitter release in neuromasts, and leads to developmental changes in synaptic ribbon size and number. These abnormalities may lead to downstream alterations in neuronal growth and patterning due to lack of sensory cell activity.

## Experimental Procedures

### Zebrafish lines and embryos

All experiments were carried out in accordance with the recommendations in the Guide for the Care and Use of Laboratory Animals of the National Institutes of Health, and in accordance with protocols approved by the Oregon State University Institutional Animal Care and Use Committee (IACUC) or the NIH Animal Care and Use program under protocol #1362-13. Tropical 5D strains of zebrafish (*Danio rerio*) were used for this study and raised on a recirculating water system (28 + 1 °C) with a 14:10 hour light-dark schedule. Spawning and embryo collection were followed as described previously^20^.

### Microinjections

Morpholinos with a sequence matching what was used in our earlier study to knock down otoferlin were injected as described previously^20^. Briefly, a pair of morpholinos (MOs) targeting the exon/intron boundaries of *otoferlin a* and *otoferlin b*, as well as a negative control MO were purchased from GeneTools, (Philomath, OR, USA). Approximately 2 nl of 0.65 mM of otoferlin a MO and 2 nl of 0.75 mM of otoferlin b MO diluted with RNAse-free ultrapure distilled water and 3% phenol red were pressure-injected in wildtype 5D embryos at one-cell stage.

### Staining with vital dyes

CLING-ATTO 488 (Synaptic Systems, Germany) was used to observe endocytosis in hair cells of zebrafish larvae^21^. Larval zebrafish at 96 hpf were anaesthetized with tricaine and incubated with 1.7 µM of mCLING diluted in embryo medium for 6 minutes at room temperature to ensure adequate probe penetration. Both control and KD zebrafish siblings were subjected to identical incubation conditions with the dye. The fish were washed with PBST for 5 minutes and fixed for 3–4 hours with 4% PFA at 4 °C. Following fixation, fish were washed with PBST at room temperature, rinsed with water and mounted with ProLong Gold Antifade Reagent (Invitrogen) for confocal imaging.

### Whole-mount Immunohistochemistry

Control and morphant zebrafish embryos were collected at 96 hpf and fixed in 4% paraformaldehyde for 3–4 hours at 4 °C. After rinsing, larvae were permeabilized with ice-cold acetone and blocked with PBS buffer containing 2% goat serum and 1% bovine serum albumin (BSA). Larvae were then incubated with primary antibodies diluted in PBS buffer for at least 2 hours at room temperature, followed by incubation with secondary antibodies (1:1000) coupled to Alexa Fluor 488, Alexa Fluor 555, Alexa Fluor 546 or Alexa Fluor 647 (Invitrogen), and mounted with ProLong Gold Antifade Reagent (Invitrogen). The following primary antibodies were used: Mouse monoclonal anti-otoferlin, Developmental Studies Hybridoma Bank, (Iowa, IA) (1:1000); Mouse monoclonal anti-Ribeye b, (Katie Kindt, National Institute of Health), (1:10,000); Mouse monoclonal anti-MAGUK, Neuromab (Davis, CA) (1:500). YOPRO-1 (Quinolinium,4-((3-methyl-2(3H)-benzoxazolylidene)methyl)-1-(3-(trimethylammonio)propyl)-,diiodide) was used to mark hair cells in zebrafish as described previously^20^.

### Confocal image acquisition and analysis

Whole-mount immunohistochemistry preparations were imaged with a Zeiss LSM 780 NLO confocal laser-scanning microscope fitted with a 63X oil-immersion objective and appropriate filters. All neuromasts were imaged using identical parameters, based on a control zebrafish neuromast, at the lowest laser power possible to prevent photobleaching. For mCLING analysis, Z-stacks were created for each neuromast, with 3–6 neuromast per 96 hpf zebrafish, beginning at the most apical portion of the neuromast and ending just beyond the basal end of the hair cells, where mCLING fluorescence appeared to stop. Care was taken to make the apical end of the neuromast at the center of the image, with dimensions of the image having only one neuromast in the frame. Prior to analysis, Average Intensity Projections were created of each z-stack, and files were converted to 16-bit JPEGs. Corrected Total Image Fluorescence (CTIF) was measured using FIJI (an ImageJ based software), where: CTIF = IntDen − (Area * Mean of background). In FIJI, the integrated density (“IntDen”) value is the product of Area of the entire image and Mean Gray Value (the average gray value within the image. First, the IntDen for the entire image was measured, and then a square was drawn in the darkest region of the image to capture the mean value for background. This method is based on previously published studies measuring fluorescence intensity with ImageJ or FIJI^22^. For Ribeye immunofluorescence image analysis, images containing Ribeye immunolabeling were corrected for background, and each image was analyzed using the ImageJ Analyze Particle option, with a size range of 0.1-Infinity µm^2^ units in each image for puncta count. This analysis software permits a relative size quantification value based on binary black or white pixels present in the image under given size range. Imaging parameters, including size and intensity were maintained the same for each image used in the assay. The total number of puncta, as well as percent of puncta within size bins 0.1–0.25, 0.25–0.5, 0.5–0.75, 0.75–1.0 and >1.0 µm^2^, were quantified for each neuromast (control N = 20, morphant N = 18).

### Calcium imaging

Calcium imaging was performed as previously described^23,24^. Transgenic (Tg) *myo6b: R-GECO1*^*vo10Tg*^ zebrafish were used for the experiment and were generated and described previously^23,24^. The hair cells in *Tg[myo6b:R-GECO1]* fish are dim in the absence of calcium and bright when bound to calcium. R-GECO1 fluorescence changes were visualized on a widefield Nikon FN1 microscope fitted with an QImaging Rolera EM-C2 EMCCD camera, a 60 × 1.0 NA Nikon CFI W Fluor water-immersion objective. Excitation was provided using a X-cite XLED1 lamp with a 505–545 nm LED using the following filter sets: excitation, 525/45 565LP, and emission, 605/70 (Chroma). Two groups were tested for the calcium imaging experiment: control injected and otoferlin depleted at 96–120 hpf. The *Tg[myo6b:R-GECO1]* fish were anesthetized in 0.04% MS-222, pinned to a Sylgard chamber, and injected with bungarotoxin directly into the heart to suppress any movement during imaging. The larval preparation was rinsed and bathed in an extracellular solution containing 140 mM NaCl, 2 mM KCl, 2 mM CaCl_2_, 1 mM MgCl_2_, and 10 mM HEPES adjusted to pH 7.3 during imaging. The lateral line hair cells were stimulated using a fluid jet to deliver a 2 s square step stimulus, and the data acquired and processed as previously described^23–25^. To quantify the R-GECO1 evoked calcium responses, in FIJI, a circular region of interest (ROI) with a diameter of 5 µm was placed on each hair cell within a neuromast. For determining the average calcium response of a single neuromast cluster, the magnitude of the response of each hair cell in a neuromast was measured and these individual responses were averaged over all measured neuromasts to obtain a per neuromast measurement. For the fluid jet evoked measurements, n = 8 neuromasts from 3 control larvae, and n = 7 from 3 otoferlin depleted larvae were used. To determine baseline intensity, the average R-GECO1 intensity during a 2 s streaming acquisition in the absence of stimulation was quantified. For determination of baseline intensity n = 13 neuromasts from 4 larvae were examined per genotype. After all live R-GECO1 measurements, larvae were fixed and immunostained for HCS-1 (Otof) as described above to ensure otoferlin depletion. Confocal images of fixed samples were acquired on the LSM 780 confocal system described below. On larvae where live, baseline R-GECO1 measurements were obtained, both fixed R-GECO1 levels and otoferlin immunolabel were imaged. Fixed R-GECO1 levels were quantified in the sample cells that were used to quantify live R-GECO1 baseline measurements.

### RNA extraction and sequencing

Total RNA from pooled microinjected control (WT), single morphant, or double morphant embryos were extracted at 96 hours post fertilization (hpf) using Quick RNA miniprep kit, (Zymo Research, CA) according to the manufacturer’s protocol. RNA concentrations were measured with NanoDrop ND-1000 UV–vis spectrophotometer (Agilent Technologies, Palo Alto, CA) and RNA integrity was analyzed with Agilent 2100 Bioanalyzer (Agilent Technologies, Palo Alto, CA). Four independent biological replicates each for control, single, and double morphant groups were prepared and submitted to the Center of Genome Research and Biocomputing (CGRB), Oregon State University, OR, USA sequencing core for library prep and 150 bp paired-end sequencing on the Illumina HiSeq3000. Sequencing reads were filtered and trimmed by running skewer on the mated fastq files based on quality score –q 30 – Q30.

### RNA seq data analysis

Paired-end sequencing reads were aligned using TopHat (v2.1.1) to the Zv9.79 zebrafish genome, only mapping reads across known splice junctions with a corresponding mate pair (–no-novel-juncs and–no-mixed parameters, respectively) and having a mate pair inner distance of 0 ± 50 bp^26^. 90–94% of the reads across all samples were successfully paired and mapped, with a mean of 22.6 million paired reads per sample. We conducted differential expression analysis with CuffDiff (v2.2.1) using the fragment bias detection and multiple read correction parameters. Sample visualization and QA/QC was performed using cummeRbund (v2.8.2) in R. Multi-dimensional scaling analysis of the FPKM distribution identified an outlier in the *otoferlin a* morphant group. As a result, we repeated our differential expression analysis in CuffDiff with the outlier sample removed. P-values were adjusted with a false discovery rate (FDR) using the Benjamini-Hochberg procedure. Transcripts were considered significantly differentially expressed with a FDR corrected *p-value* ≤ 0.05 and a fold change of ≥1.5. We visualized the double morphant transcription profile using a bi-hierarchically clustered heatmap with a custom R script. Fold-change values were calculated for each replicate by dividing the sample FPKM value by the average control FPKM value for each transcript. Sample and transcript clustering was performed using the agglomerative complete-linkage clustering algorithm based on a Euclidean distance matrix of the FPKM fold-changes.

### Quantitative RT-PCR

Total RNA was extracted from control (WT) and otoferlin double morphant embryos collected at 96 hpf with RNAzol (Molecular Research Centre, OH, USA) and cDNA was synthesized using High-Capacity cDNA Reverse Transcription Kit (Applied Biosystems). Primers for monitoring gene expression changes were designed from genomic sequences found in Ensembl. Extracted RNA from different biological replicate groups were used to validate expression changes. All qRT-PCR assays were performed in a 20 μl reaction volume consisting of 10 μl Power SYBR Green PCR master mix (Applied Biosystems), 0.4 μl each primer, 7.2 μl H_2_O and 50 ng equivalents of cDNA on the 7500 Fast Applied Biosystems machine. Relative fold change values in otoferlin double morphants compared to injected controls were calculated for genes of interest and normalized to *beta-actin*.

### RT-PCR of the rescue experiments

Full length mouse otoferlin (denoted as FL, amino acids 1–1992) was cloned into the mCherry-SEpHluorin vector (Addgene plasmid # 32001). For rescue experiments, the construct was co-injected with the morpholinos as previously described, and the larvae were screened for rescue of the hearing and balance phenotype^20^. Screened larvae were subsequently probed by qRT-PCR for restoration of gene expression.

### Statistics

Data were analyzed with the Prism software version 5.0 or Sigmaplot 11, with a significance value set at p ≤ 0.05. For mCLING studies, differences in CTIFs values for controls and double KDs were assessed using one-way analysis of variance (ANOVA) within groups and t-test between groups. For Ribeye immunofluorescence, a t-test was used to compare the average number of Ribeye puncta, average Ribeye puncta size, and average percent of Ribeye puncta within a size bin between control and double KD neuromasts. For intracellular calcium experiments, differences in the magnitude of the mechanically-evoked R-GECO1 calcium responses between control and double KDs, as well as differences in the baseline R-GECO1 of control and double KDs, were analyzed using t-test. For qRT-PCR data, t-tests were used to compare control and double KDs within a single developmental time period, with the exception of the gene rescue studies, where a one-way analysis of variance test was applied to test for significant differences between control, double KD, and double KD + (m)*Otof*.

## Results

### Otoferlin depleted hair cells display reduced synaptic vesicle recycling

**Figure 1:**
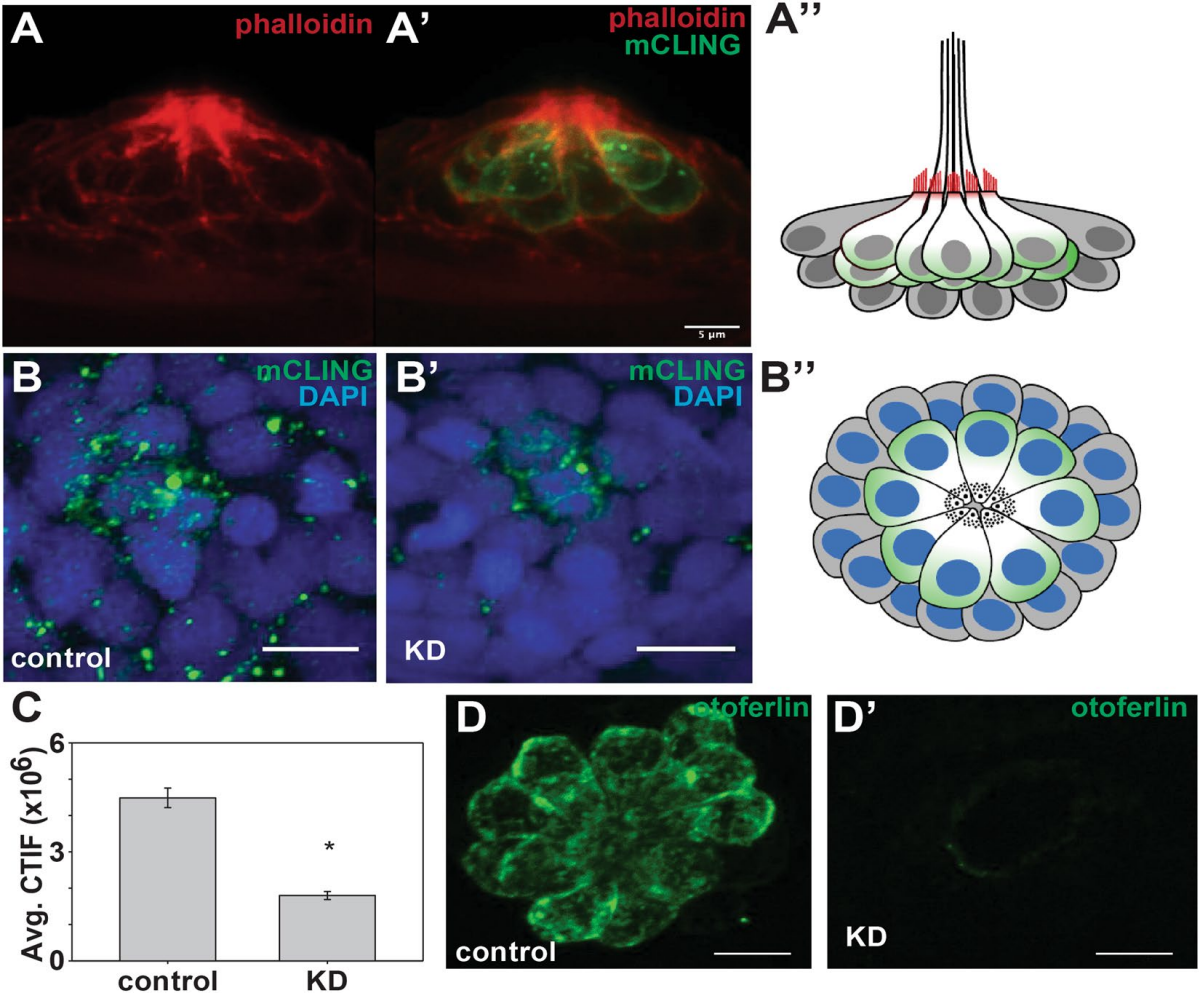
Depletion of otoferlin results in reduced hair cell vesicle recycling. (**A**–**A**’) Representative confocal images of 96 hpf (**A**) phalloidin stained neuromasts and (A’) phalloidin and mCLING stained neuromast viewed laterally. (**A”**) A diagram of a neuromast viewed laterally, with phalloidin stained actin denoted in red, and mCLING loaded hair cells denoted in green. Support cells are depicted in grey. (**B**–**B**”) Representative confocal images of control morpholino injected (**B**) and otoferlin morphant injected (**B**’) lateral line neuromasts labeled with DAPI (blue) and mCLING (green) of mCLING incubation at room temperature. (**B**”) A diagram of a neuromast in the same orientation as B, B’, with DAPI denoted in blue, and mCLING loaded hair cells denoted in green. Support cells are depicted in grey. (**C**). Quantification of average mCLING dye associated with neuromasts of negative control injected and morphant zebrafish (t-test, *p* < *0*.*001*). N = 6 larvae, 3–6 neuromasts per larvae for both negative control and morphant. (**D**–**D**’) Representative images of lateral line neuromasts stained for otoferlin (green) under (**D**) control injected and (**D**’) morpholino injected conditions. Scale bars = 5 µm.

Zebrafish lateral line neuromast hair cells are structurally and morphologically similar to mammalian type II vestibular hair cells, and possess synaptic ribbons that abut the active zone of the presynaptic membrane where synaptic vesicles dock in preparation for exocytosis. Previously we reported that the C2 domains of otoferlin both bind calcium and enhance liposome fusion, and that depletion of otoferlin in zebrafish larvae resulted in a ‘circler’ phenotype whereby larvae are defective in hearing and balance and fail to develop an inflated swim bladder^16,20^. Thus, it appears that loss of otoferlin in zebrafish partially phenocopies the mouse knockout model, though we did not previously verify that otoferlin depletion resulted in attenuated synaptic vesicle recycling^20^. To affirm that, like the mouse knockout model, loss of otoferlin in zebrafish disrupts synaptic vesicle recycling, we monitored mCLING loading in control and double knockdown neuromasts (Fig. 1). mCLING is a fluorescent dye with selective uptake via vesicle endocytosis^21^. After verifying the mCLING is internalized by hair cells (Fig. 1A–A”), we quantified mCLING loading for neuromasts of age-matched control and morphant zebrafish (Fig. 1B,C). We found that otoferlin depleted neuromasts were associated with significantly less mCLING (Fig. 1C, Supplemental Fig. 1) when compared to control zebrafish (t-test, p < 0.001), suggesting reduced or defective vesicle recycling in agreement with previous observations in a mutant mouse model^21^. There was no significant difference in the number of optical slices per z-stack (t-test, p = 0.1746). We also found no significant differences in total image intensity within the control zebrafish, nor the KD zebrafish (One-way ANOVA). Overall our results are similar to an mCLING study using a mouse model which found reduced mCLING internalization in hair cells lacking otoferlin^21^.

### Otoferlin depletion changes the architecture of the ribbon synapse

**Figure 2:**
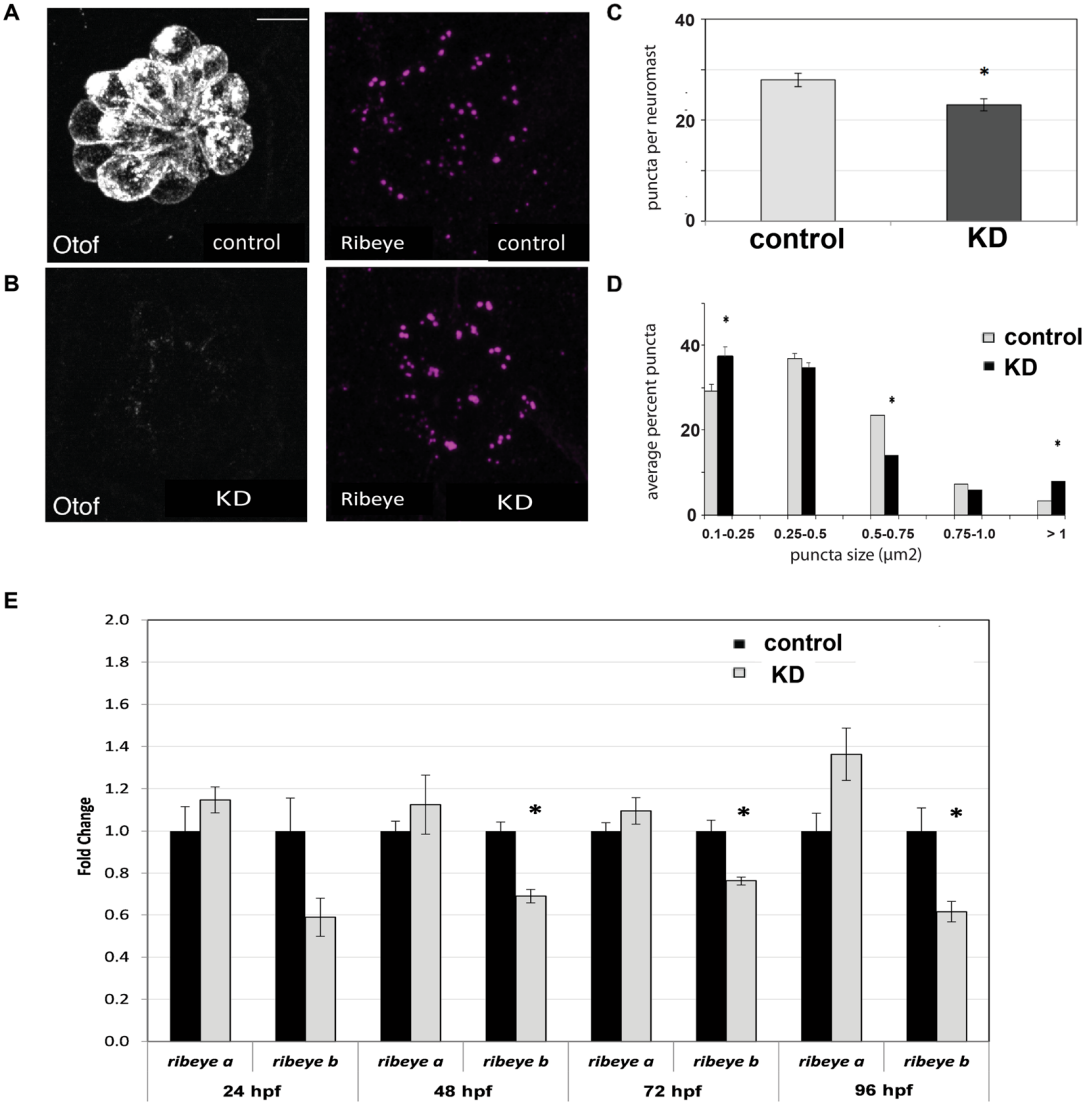
Effects of otoferlin depletion on synaptic morphology in posterior lateral line neuromasts of 96 hpf zebrafish larvae. (**A**,**B**) Representative confocal z-projection images of neuromasts from control injected (**A**) and otoferlin morphant (**B**) larvae immunolabeled with anti-otoferlin (HCS-1), and anti-Ribeye. (**C**) Average number of Ribeye puncta counted per neuromast in negative control injected (no. of neuromasts = 20) and morphant (no. of neuromasts = 18) larvae (t-test, *p-value* < *0*.*0001)*. Error bars indicate s.e.m. (**D**) Average percent of Ribeye puncta per neuromast that fell within size bins of 0.1–0.25, 0.25–0.5, 0.5–0.75, 0.75–1.0, and >1.0 µm^2^, in control injected (no. of neuromasts = 20) and morphant (no. of neuromasts = 18) larvae (t-test, *p-value* < *0*.*0001)*, Error bars indicate s.e.m. (**E**) Expression of ribeye transcripts in control and morphant larvae at 24, 48, 72, and 96 hpf, N = 4 per time point (t-test, *p-value* < 0.05). Scale bars = 5 µm.

We next sought to examine the morphology of the hair cell ribbon synapse by imaging Ribeye immunolabeled lateral line neuromasts in larvae 96 hours post fertilization (hpf). We observed that otoferlin depleted hair cells displayed an atypical distribution and number of Ribeye puncta relative to control (Fig. 2A–E). The average number of Ribeye puncta per neuromast was significantly fewer in otoferlin depleted larvae as compared to control (Fig. 2C).

In addition, a quantitative comparison of puncta revealed a difference in the size distribution of Ribeye puncta in otoferlin depleted larvae relative to age-matched controls. Morphants neuromasts had a significantly larger percentage of “larger” Ribeye puncta, as defined by the puncta size bins (Fig. 2D). These large Ribeye puncta may represent single, large synaptic ribbons, or alternatively, clusters of ribbons that could not be visually resolved (Supplemental Fig. 2). In agreement with our previous study, no change in the number of hair cells per neuromast was found in the otoferlin depleted larvae, as determined previously using YOPRO-1 (data not shown)^20^. In addition, Ribeye puncta appeared to localize proximal to MAGUK in both control and morphant neuromasts (Supplemental Fig. 3). To quantitate mRNA levels of ribeye, we conducted qRT-PCR at 24, 48, 72 and 96 hpf. We found that expression of *ribeye b* was significantly lower in morphants compared to controls at 48, 72 and 96 hpf (Fig. 2E), suggesting that loss of otoferlin affects both the assembly of the synaptic ribbon and Ribeye b expression.

**Figure 3:**
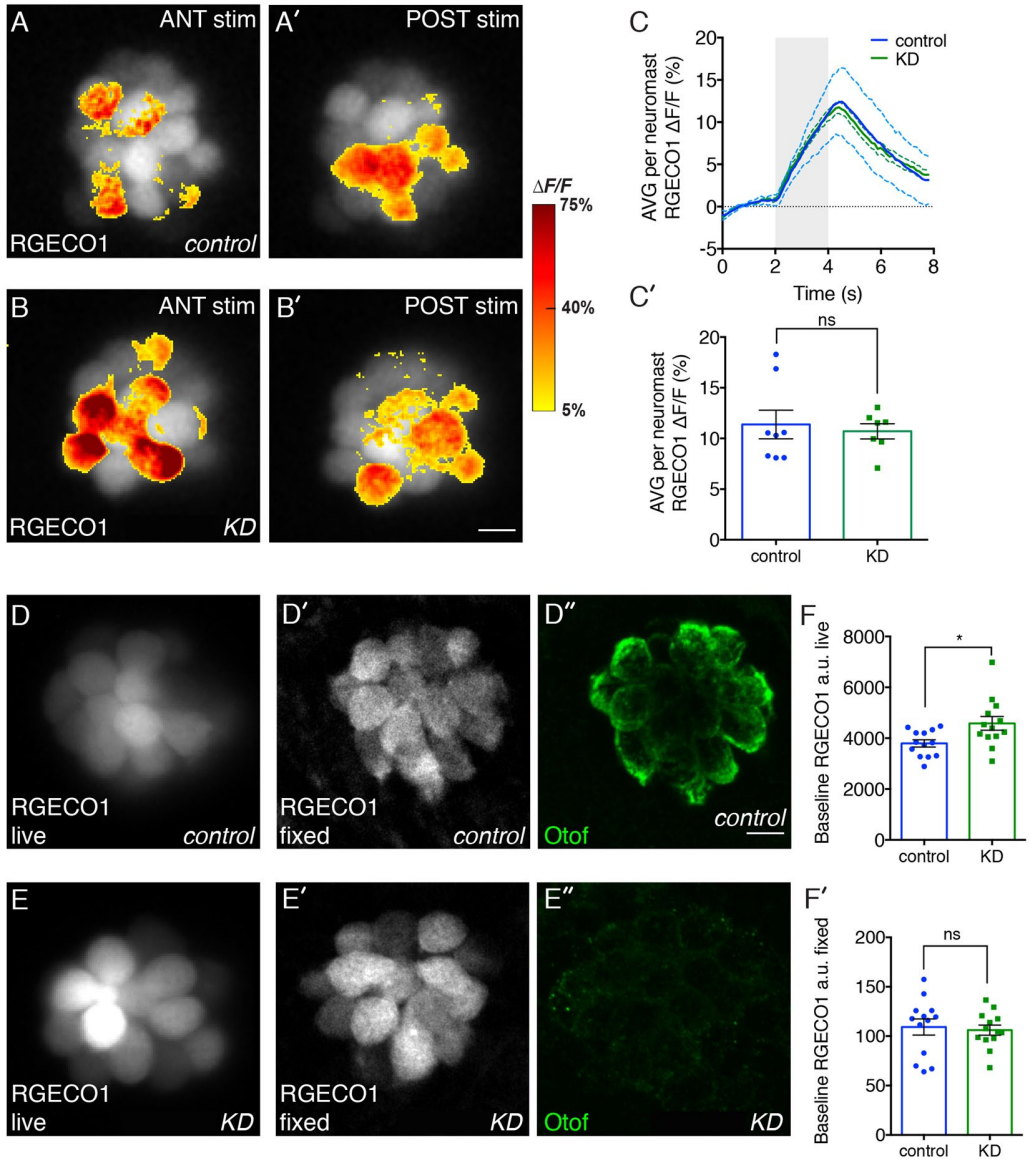
Effects of otoferlin depletion on intracellular calcium in posterior lateral line neuromasts. Representative evoked R-GECO1 calcium signals in negative control injected (**A**–**A**’) and otoferlin depleted (**B**–**B**’) neuromasts during mechanical stimulation of hair bundles. The ANT (anterior) and POST (posterior) responses reflect the two directions the posterior lateral-line neuromasts are mechanically sensitive. Calcium signals during stimulation (**A**–**A**’ and **B**–**B**’) are colorized according to the heat map and superimposed onto baseline images. (**C**,**C**’) The magnitude of the mechanically-evoked R-GECO1 calcium responses were not significantly altered between control and otoferlin depleted neuromasts, t-test p = 0.70, n = 8 control and 7 KD neuromasts. (**D**,**E**) The live, R-GECO-1 baseline intensity in otoferlin depleted hair cells was significantly elevated compared to controls (**D**–**F**) t-test, p = 0.016. In the same samples as (**F**), the fixed, R-GECO1 levels (**D**’,**E**’) were not significantly different in otoferlin depleted hair cells compared to controls (**F**’), t-test, p = 0.745, n = 13 neuromasts. (**D**”,**E**”) Samples were also immunostained with HCS-1 (Otof) to ensure depletion was effective. Scale bars = 5 µm.

### Otoferlin depleted sensory hair cells display abnormal intracellular calcium handling

**Figure 4:**
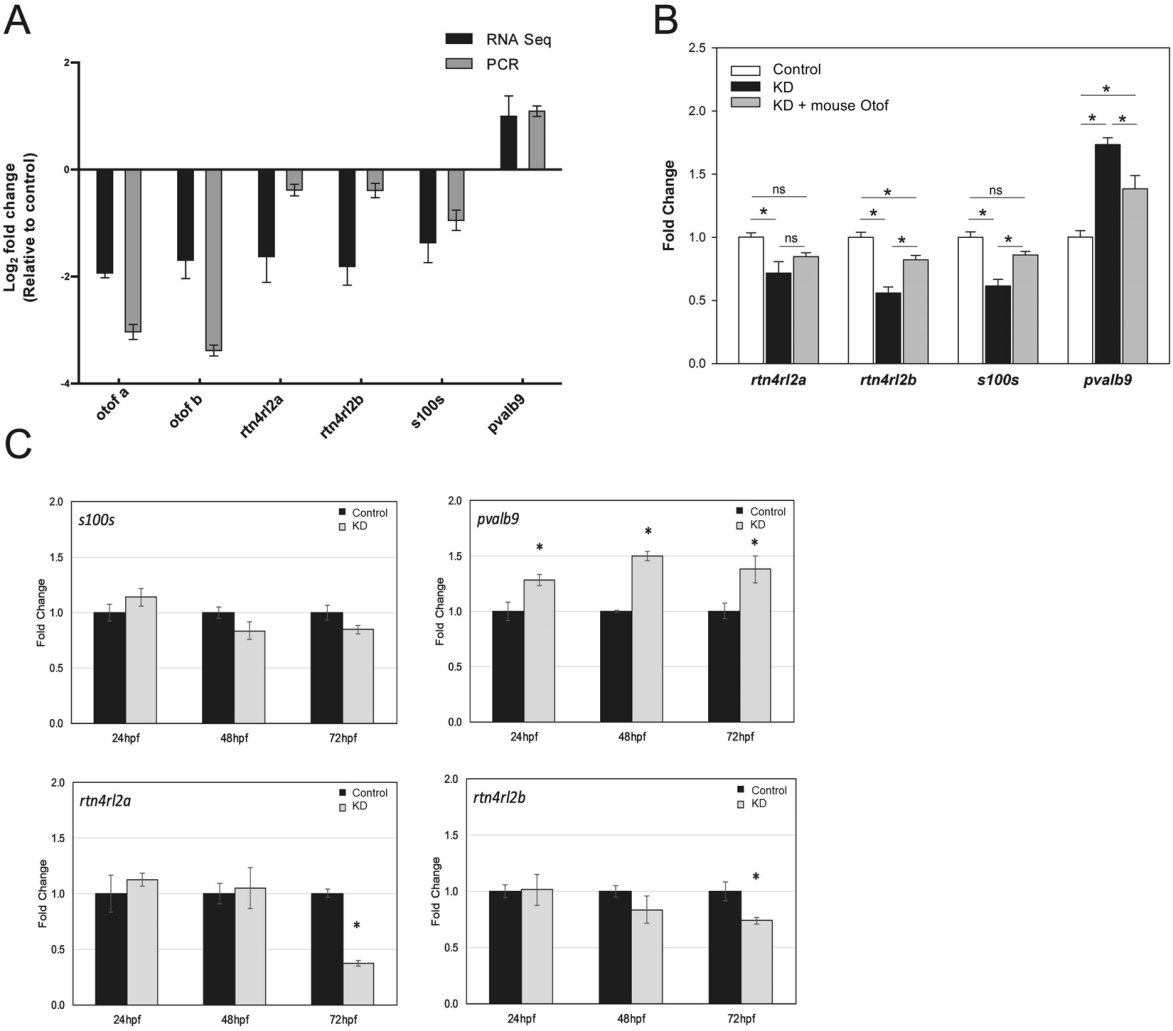
RNA sequencing and RT-qPCR for differentially expressed genes in otoferlin depleted larvae. (**A**) Black bar graphs represent relative mRNA expression changes of the upregulated and downregulated genes derived from transcriptomic analysis (no. of biological reps. =4, each replicate consists of 15 pooled embryos). Grey bars represent qRT-PCR data of mRNA expression changes of genes in larval otoferlin mutant zebrafish at 96 hpf (no. of biological reps. = 4, each replicate consists of 30 pooled embryos). Each gene is normalized with respect to the corresponding controls. qRT-PCR, ΔΔCt values were calculated by comparing ΔCt values (normalized to beta-actin) to the mean ΔCt for each gene. Data were analyzed by Wilcoxon’s test with standard 5% significance level. Abbreviations *otof a*: Otoferlin a, *otof b*: Otoferlin b, *rtn4rl2a*: Reticulon 4 Receptor like 2a, *rtn4rl2b*: Reticulon 4 Receptor like 2b, *s100s*: S100 Calcium Binding Protein S, *pvalb9*: Parvalbumin 9. (**B**) Rescue of repressed transcripts in otoferlin depleted larvae. Bar graphs represent relative mRNA expression changes of transcripts in otoferlin depleted larvae at 96 hpf (denoted otoAB KD, black bars) and rescue of the transcripts (denoted (m) otof, grey bars) in morphants coinjected with full length otoferlin rescue construct. Each gene is normalized with respect to the corresponding controls (white bars). ΔΔCt values were calculated by comparing ΔCt values (normalized to beta-actin) to the mean ΔCt for each gene. Data were analyzed by t-test. For *pval9b* and *rtn4rl2b* groups both otoferlin depleted and rescue samples showed statistically significant changes in expression compared to controls (p-value < 0.05). For *rtn4rl2a* significant repression in expression for the KD were found (p-value < 0.05), and there was restoration of repressed transcripts in the rescue larvae. (**C**) Expression of *s100s*, *pval9b*, *rtn4rl2a*, *rtn4rl2b* at 24, 48 and 72 hpf in control and otoferlin depleted zebrafish larvae (n = 4 p < 0.05). For (**A**–**C**), error bars are s.e.m.

Cav1.3a zebrafish mutants develop an enlarged synaptic ribbon similar to the enlarged Ribeye puncta we observed in otoferlin depleted hair cells^24^. In addition to enlarged ribbons, the Cav1.3 zebrafish mutants also displayed a loss of L-type calcium channel activity in response to mechanical deflection of stereocilia. To determine if otoferlin depletion affects intracellular calcium, we measured calcium transients in control and otoferlin depleted neuromasts using a transgenic line that expresses R-GECO1 (Red fluorescent - Genetically Encoded Calcium indicator for Optical imaging) in hair cells. R-GECO1 is derived from GCaMP3 and mApple and allows detection of calcium influx in R-GECO1 expressing hair cells via an increase in fluorescence when bound to calcium. To examine intracellular calcium levels, we first examined calcium signals during mechanical deflection of the hair bundles. We did not observe significant differences in the evoked calcium responses between control and otoferlin depleted hair cells (Fig. 3A–C’), indicating that otoferlin depleted hair cells have intact hair bundles and exhibit normal mechanosensation. However, we did observe that the resting calcium level in hair cells was higher in otoferlin morphants relative to control larvae (Fig. 3D–F). After taking live, resting calcium measurements, we fixed larvae and measured the levels of R-GECO1 in our samples. We found that the amount of R-GECO1 in our fixed hair cells was not different between otoferlin morphants and control larvae (Fig. 4F’). This suggests that our live R-GECO1 intensity measurements reflect differences in resting calcium rather than differences in R-GECO1 expression levels. Overall our calcium measurements using R-GECO1 indicate that while evoked calcium signals are normal in otoferlin depleted hair cells, the resting calcium levels are significantly elevated in otoferlin morphants relative to controls.

### Otoferlin depletion alters the transcriptome of zebrafish

The observed difference in resting calcium suggests loss of otoferlin may change expression of genes associated with intracellular calcium. To test for changes in global gene expression, we conducted 150 bp RNA-sequencing and subsequent qRT-PCRs on whole 96 hpf control and otoferlin depleted zebrafish. Otoferlin depletion resulted in significant differential expression of 433 transcripts, of which 271 were decreased and 162 were increased (FDR adjusted P-value ≤ 0.05; fold change ≥1.5, Supplemental Fig. 4). Both otoferlin genes (*otof a* and *otof b*) were significantly decreased with log2 fold changes of −1.93 and −1.69 respectively, confirming the depletion of otoferlin. Of the transcripts altered in the otoferlin depleted larvae, we selected those associated with neuromasts that displayed the greatest fold changes and validated the expression change by qRT-PCR. Analysis of the qRT-PCR results matched those of the RNAseq results for all transcripts tested (Fig. 4A). Several transcripts play critical roles in morphogenesis and maturation of the lateral line and otic region (*rtn4rl2a*, *rtn4rl2b*), and several are associated with calcium related maintenance of lateral line hair cells (*s100s*, *pvalb*9)^12,27–32^. To ensure that the changes in expression are due to otoferlin depletion, we co-injected morpholinos with an exogenous otoferlin expression vector and monitored expression of the identified genes (Fig. 4B). The expression vector harbors the coding sequence of mouse otoferlin, which is not recognized by the morpholinos. We found that co-injection resulted in expression levels of *rtn4rl2a, rtn4rl2b, pvalb9*, that were partially restored to control levels, and restoration of *s100s* to levels indistinguishable from controls (Fig. 4B). We conclude that the observed changes in expression for these genes were otoferlin-sensitive. Finally, to determine the onset of abnormal expression in morphants, we measured expression of *rtn4rl2a*, *rtn4rl2b*, *pvalb9*, and *s100s* at 24, 48, and 72 hpf (Fig. 4C). We found down regulation of both *rtn4rl2a* and *rtn4rl2b* at 72 hpf, while *pvalb9* was significantly upregulated at all three time points measured in otoferlin depleted conditions (Fig. 4C). Significant changes in *s100s* were not detected between 24–72 hpf. The lack of a significant change in *s100s* transcript levels in the three days prior to a down regulation at 96 hpf may be a result of the relatively low number of s100-positive cells present in the animals earlier in development as the number of s100-positive cells in neuromasts is known to increase with age.

## Discussion

Overall the major findings of this study are that otoferlin depletion leads to deficits in vesicle recycling, changes in the synaptic ribbon, abnormal calcium baseline levels, and altered gene expression. The observed attenuation of internalized mCLING is consistent with a previous report of reduced mCLING loading in a *Otof*^*−/−*^ mouse model^21^. It has been proposed that otoferlin functions in synaptic vesicle priming and exocytosis, and may also contribute to exocytosis-endocytosis coupling^17,33,34^. The reduction in mCLING internalization in zebrafish supports this proposed function, and suggests a conserved role for otoferlin in vertebrates. A conserved function for otoferlin is also supported by the finding that human and zebrafish otoferlin are approximately 75% identical in amino acid sequence despite 400 million years divergence between these organisms^20^. Thus, it is likely that conclusions based on our observations in zebrafish will hold true for other vertebrates, including mammals.

Analysis of our immunostaining measurements indicated that otoferlin depleted hair cells displayed abnormal synaptic ribbons. Otoferlin depletion resulted in fewer, but larger ribbons, although we could not discern between single enlarged synaptic ribbons and multiple closely-spaced ribbons with distances less than can be resolved using confocal microscopy. In mice, loss of otoferlin also leads to synaptic ribbon abnormalities, with both an increased number of floating ribbons and more numerous ribbon anchoring deficiencies^15^. In nascent hair cells, Ribeye is detected as small particles throughout the cytoplasm, but upon cell maturation forms larger structures along the basolateral membrane^11,35^. One possible explanation for abnormal ribbon formation may be due to the loss of neurotransmitter release from otoferlin depleted hair cells. This seems unlikely however, since loss of neurotransmitter release from VGlut3 mutant zebrafish hair cells does not lead to abnormal synaptic ribbons^36^. We also observed lower levels of *ribeye b* transcript expression in otoferlin depleted larvae. We propose that the decreased *ribeye b* expression, at least in part, accounts for the observed abnormalities.

In addition to synaptic ribbon abnormalities, otoferlin depletion resulted in elevated R-GECO signal in resting sensory hair cells, suggesting that reduced expression of otoferlin may alter baseline calcium levels. In addition, expression of the calcium buffer protein *pvalb9* and the calcium signaling protein *s100s* were altered under otoferlin depletion. The use of zebrafish afforded us the opportunity to easily and quickly conduct rescue experiments which are significantly more difficult to perform in mouse models. Transcript levels of both *pvalb9* and *s100s* were restored when exogenous otoferlin was expressed in larvae depleted of endogenous otoferlin. Thus, we speculate that otoferlin depletion may alter calcium signaling in the sensory hair cells. However, our R-GECO measurements did not reveal significant deficiencies in calcium influx during stimulation, suggesting that flow through the Cav1.3 channels are not the source for any disruption in calcium signaling.

We also find that loss of otoferlin results in changes in expression of genes associated with both neuronal growth and the development and maintenance of sensory hair cells. Specifically, we observed significant reductions in the expression of Nogo receptor homologs *rtn4rl2a* and *rtn4rl2b*. Nogo-A/Rtn4a belongs to the neurite outgrowth inhibitory family of signaling protiens^37^. We speculate that these changes in transcripts represent an attempt to compensate for loss of signaling between the hair cell and afferent neuron. Early developmental differences in neuronal growth owing to knockdown provide a possible explanation for the observation that re-expression of endogenous otoferlin upon loss of morpholino potency does not restore hearing and balance in zebrafish^20^. Changes in the expression of neuronal genes upon loss of signaling from sensory hair cells also provides a molecular basis for a recent study that reported a reduction of auditory nerve and ventral cochlear nuclei size in otoferlin mutant mice^38^.

Our conclusions are based on morpholino knockdown of otoferlin expression. We have previously determined that expression of exogenous otoferlin rescues the deafness and balance defects associated with endogenous otoferlin depletion^20^. In this study, we have demonstrated that exogenous expression rescues expression of genes affected by knockdown. We therefore conclude that while nonspecific effects are possible, it appears that the observed phenotype is not the result of off-target effects.

Additional studies are required to elucidate the relationship between otoferlin, intracellular calcium, and the identified otoferlin-sensitive calcium signaling proteins (s100s, pvalb9). Future studies should also focus on establishing a mechanism for the observed alteration of ribbon synapses in response to loss of otoferlin.

## Supplementary information

Supplemental Figures
