# Supplementary material for: Otoferlin Depletion Results in Abnormal Synaptic Ribbons and Altered Intracellular Calcium Levels in Zebrafish
Source: Sci Rep. 2019 Oct 3;9:14273. doi: 10.1038/s41598-019-50710-2 (PMC6776657; doi:10.1038/s41598-019-50710-2)
Supplement: Supplementary file 1 — Supplemental Figures [file 41598_2019_50710_MOESM1_ESM.docx]

**Supplemental Material**

**Otoferlin Depletion Results in Abnormal Synaptic Ribbons and Altered Intracellular Calcium Levels in Zebrafish**

Aayushi Manchanda 1, #, Paroma Chatterjee 1, #, Josephine A. Bonventre2, Derik E. Haggard 3, Katie S. Kindt 4, Robert L. Tanguay 1, 3, Colin P. Johnson 1, 2*

1Molecular and Cellular Biology Program, 2Department of Biochemistry and Biophysics, 3Department of Environmental and Molecular Toxicology, Oregon State University, Corvallis, Oregon, USA; 4National Institute of Deafness and Other Communication Disorders (NIDCD), NIH, Maryland, USA. # These authors contributed equally.

Supplemental Figure 1


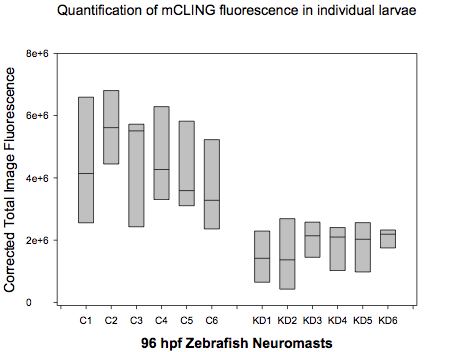


**mCLING quantitative analysis:** Z-stacks were created for each neuromast, with 3-6 neuromast per 96 hpf zebrafish, beginning at the most apical portion of the neuromast and ending just beyond the basal end of the hair cells, where mCLING fluorescence appeared to stop. Z-stacks were on average 20.6 (± 6.5) slices, and there was not a significant difference in the number of slices per z-stack for control and KD neuromasts (t-test, p = 0.1746). Care was taken to make the apical end of the neuromast at the center of the image, with dimensions of the image had only one neuromast in the frame. Prior to analysis, Average Intensity Projections were created of each z-stack, and files were converted to JPEGs. Corrected Total Image Fluorescence (CTIF) was measured using ImageJ (FIJI), where: CTIF = IntDen –(Area * Mean of background). In ImageJ/FIJI, the integrated density (“IntDen”) value is the product of Area of selection in square pixels (in this case the entire image) and Mean Gray Value (the average gray value within the selection). First, the IntDen for the entire image was measured, and then a square was drawn in the darkest region of the image to capture the mean value for background. No significant difference was observed between neuromast CTIFs for each zebrafish within the two groups (One-way ANOVA).

Supplemental Figure 2:

**Ribeye Puncta Distribution:** Representative image of Ribeye stained neuromast. Boxed region is shown enhanced on the right. Scale bar = 5µm.

Supplemental Figure 3:


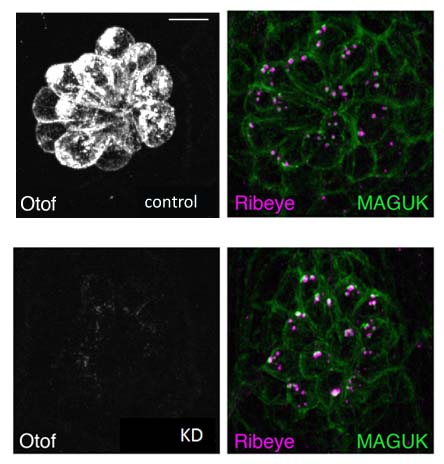


**Ribeye localizes proximal to MAGUK:** (A) Representative images of control injected (top panels) and otoferlin morphant (bottom panels) showing otoferlin (left, denoted Otof), and (right panels) Ribeye (purple) and MAGUK (green) costained neuromasts. Scale bar = 5µm.


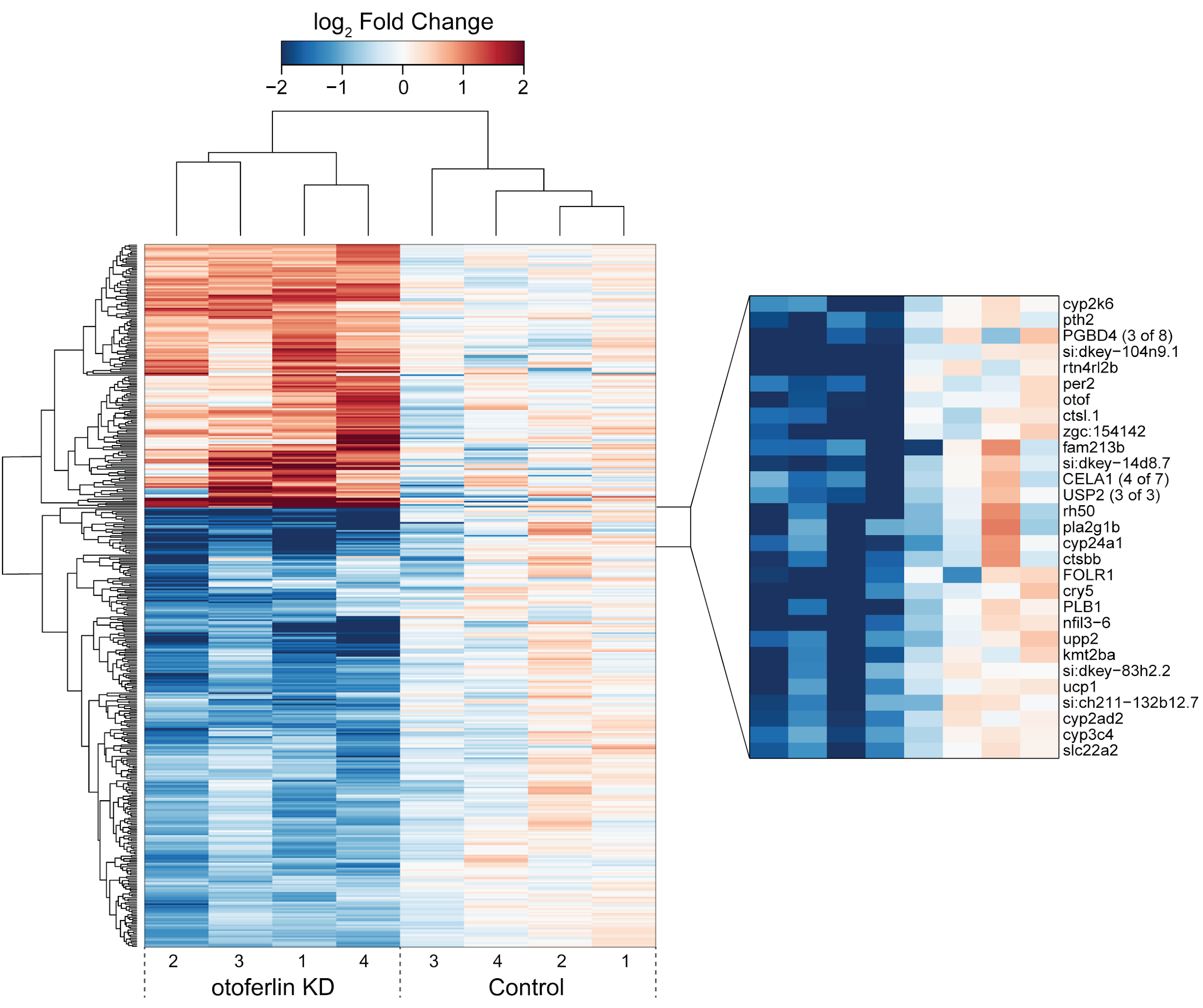
Supplemental Figure 4:

**Heat Map of RNAseq data sets for 96 hpf control injected and morphant larval zebrafish.** Bi-hierarchically clustered heatmap of the 433 significantly differentially expressed transcripts in otoferlin depleted larvae (FDR adjusted P-value ≤ 0.05; fold change ≥ 1.5). The inset on the right highlights the cluster of the most highly decreased transcripts.
